# Comparison of human papillomavirus (HPV) detection in urine and cervical swab samples using the HPV GenoArray Diagnostic assay

**DOI:** 10.7717/peerj.3910

**Published:** 2017-10-09

**Authors:** Pornjarim Nilyanimit, Jira Chansaenroj, Anant Karalak, Piyawat Laowahutanont, Pairoj Junyangdikul, Yong Poovorawan

**Affiliations:** 1 Center of Excellence in Clinical Virology, Faculty of Medicine, Chulalongkorn University, Bangkok, Thailand; 2 Department of Pathology, National Cancer Institute, Bangkok, Thailand; 3 Department of Gynecology, National Cancer Institute, Bangkok, Thailand; 4 Department of Pathology, Samitivej Srinakarin Hospital, Bangkok Hospital Group, Bangkok, Thailand

**Keywords:** HPV, Urine, Genotyping, Cervical cancer

## Abstract

Human papillomavirus (HPV) is the leading cause of cervical cancer. Urine-based HPV testing offers a simple and non-invasive method because of its increasing acceptance. A total of 164 pairs of cervical swab and urine samples from Thai women who underwent cervical cancer screening were used for HPV testing with HPV GenoArray Diagnostic Kits. The overall concordance percentage for HPV detection in the cervical swab and urine samples was 65.2%. The HPV genotypes most commonly detected were HPV16 and HPV18. An analysis of the urine samples and a second analysis of the cervical swab samples showed that the differences in the overall HPV detection rate between women with normal and abnormal cytology were not significant (*p* > 0.05). Urine samples processed with the GenoArray assay is an alternative for women who decline to undergo Pap smear even though it is not ideal as the first-line screening option.

## Introduction

Human papillomavirus (HPV) causes cervical cancer (Koutsky, 1997). Approximately 170 genotypes have been identified (De Villiers et al., 2004) and at least 40 genotypes infect the human anogenital tract (De Villiers, 2013). The genital HPVs are classified into high-risk and low-risk genotypes depending on their association with uterine cervical cancer (Muñoz et al., 2003). The high-risk genotypes most commonly detected in uterine cervical cancer are HPV16, 18, 31, 33, 35, 45, 52, 58, 39, 51, 56, and 59 (Bouvard et al., 2009).

The incidence of cervical cancer in young women is increasing in many countries due to improved awareness and testing. HPV prevalence in Asia, Africa, Europe and South America varied significantly depending on the population and geographical regions (Clifford et al., 2005). In Europe, it ranges from 1.4% (Spain) to 9.2% (Italy). In South America, the prevalence ranged from 11.9% (Chile) to 16.3% (Argentina). In Southeast Asia, the prevalence ranged from 1.6% (Vietnam) to 13.3% (Korea). Even within a country such as Thailand, HPV prevalence can vary from 7.2% in northern region to 15.1% in central region (Kantathavorn et al., 2015).

The Papanicolaou (Pap) test is a cost-effective way to screen for cervical cancer. Test results help physicians detect precancerous lesions and determine the course of treatment. Pap test has been shown to reduce the incidence of mortality from cervical cancer (Mählck, Jonsson & Lenner, 1994). However, it is primarily used for detecting invasive cervical cancer and cannot identify asymptomatic HPV infection (Safaeian, Solomon & Castle, 2007; Leyden et al., 2005). In addition, barriers to testing include the lack of information, personal preference, fear, embarrassment and lack of trust in healthcare under certain circumstances. False information regarding the procedure, the lack of spousal support towards screening, cultural taboos, and stigmatization of women with cervical cancer further contribute to the limitations of the Pap test. Therefore, alternative and supplementary HPV DNA assays are often required in combination with the traditional Pap smear test (Cox et al., 1995).

HPV DNA detection in urine samples presents a feasible alternative to HPV DNA detection in cervical specimens. Urine testing provides an especially simple, non-invasive method for screening (Prusty et al., 2005). The benefits of using urine for HPV DNA detection have been evaluated in disease surveillance and screening for cervical cancers involving specific genotypes. HPV DNA urine testing can be used to identify abnormal cells in adolescent girls and young women who do not wish to have a vaginal examination (Vorsters et al., 2014; Enerly, Olofsson & Nygård, 2013). Some studies have reported a high HPV detection sensitivity for urine-based assays (Forslund et al., 1993; Hagihara et al., 2016; Bernal et al., 2014), while other studies have reported a low HPV detection sensitivity from urine-based assays (D’Hauwers et al., 2007; Nilyanimit et al., 2013).

Molecular methods for HPV testing have been explored, such as PCR/sequencing (De Roda Husman et al., 1995), the INNO-LiPA HPV Assay (Van Hamont et al., 2006), and the Hybrid Capture 2 test (HCII) assay (Kubota et al., 1998). In addition, the HPV GenoArray Diagnostic Kit (Hybribio Ltd., Sheung Wan, Hong Kong) is a recently developed PCR-based HPV genotyping assay, which uses L1 consensus primers to amplify 21 HPV genotypes. It is then followed by flow-through hybridization with immobilized genotype-specific probes. This test is currently used in several hospitals in China (Liu et al., 2010). A previous study showed that the sensitivity of the HPV GenoArray assay was 97.8% and the specificity was 100% (Du et al., 2013). If its use expanded to other parts of Asia where individuals share similar cultural beliefs, more women would benefit from increased cervical cancer screening. This study aimed to evaluate the use of a urine-based assay as a non-invasive method for HPV detection and to genotype the samples using the HPV GenoArray assay in a Thai population.

## Materials & Methods

The research protocol was approved by the Ethics Committee of the National Cancer Institute, Thailand (number EC COA 037/2012), and the Institutional Review Board of the Faculty of Medicine at Chulalongkorn University (number 389/2555). The objective of the study was explained to the patients, and written consent was obtained from all participants. Each specimen was sent for testing anonymously, which only included participant-specific numerical code and age information.

### Clinical specimens

All patients underwent the Pap smears test and were subsequently asked to participate in this study. In all, 164 women consented and were willing to provide paired specimens (a Pap smear sample from the cervix and a first-void urine sample). All specimen were recruited between March to December 2014. Specimens were classified into three groups: 95 samples indicating normal cytology, 50 samples indicating low-grade squamous intraepithelial lesions (LSIL), and 19 samples indicating high-grade squamous intraepithelial lesions (HSIL). The ages of the patients enrolled in this study were between 19 and 69 years.

### Sample preparation

Each Pap smear sample (which are the standard samples for HPV genotyping) was evaluated by a specialized cytotechnologist and the results were confirmed by a pathologist. Samples were suspended in the liquid-based cytology (LBC) buffer (ThinPrep, Hologic, Marlborough, MA, USA). Collection of the first-void urine (FVU) samples was performed in a sterile Cell PrepPlus (Biodyne, Gyeonggi-do, Korea) urine bottle, stored at 4 °C, and processed within 3 days. For each sample, approximately 15 mL of LBC and FVU were centrifuged at 3,000 rpm for 5 min, and the supernatant was removed. The residual 800 µL of the sample suspension containing cell debris was washed and centrifuged at 8,000 rpm for 5 min. The DNA was extracted from the pellets using a DNA Prep Kit (Chaozhou Hybribio Biochemistry Ltd., Guangdong, China) and stored at −20 °C until testing.

### HPV GenoArray Diagnostic assay

The extracted DNA from the cervical swab and urine samples was subjected to an HPV genotyping assay using HPV GenoArray Diagnostic Kits (Hybribio Ltd., Sheung Wan, Hong Kong) according to the manufacturer’s instructions. This PCR-based assay enables the amplification of 21 HPV genotypes including 13 high-risk types (HPV 16, 18, 31, 33, 35, 39, 45, 51, 52, 56, 58, 59, and 68), two probable high-risk types (HPV 53 and 66), and six low-risk or unknown risk types (HPV 6, 11, 42, 43, 44, and CP8304 [HPV-81]). This assay uses an L1 consensus primer-based PCR and is different from the Linear Array HPV Genotyping Test, which uses MY09 and MY11 primers (Liu et al., 2010). After PCR amplification, the amplicons were subjected to flow-through hybridization on a nylon membrane covered in immobilized HPV genotype-specific oligonucleotide probes. The hybrids were detected by the addition of streptavidin-horseradish peroxidase conjugate and substrate (NBT/BCIP). The presence of a positive result for the internal control and the biotin dots within the membrane serves to validate DNA quality, good enzyme conjugate, and successful hybridization process. Results were manually interpreted using the manufacturer’s guidelines. The normal detection limit is ∼500 copies/µL of target HPV DNA. There were no cross-reactivities from the amplification/detection of the 21 HPV genotypes. The provided positive control and two negative controls (including HPV-negative C33-A cells) were included in each set of PCR to assess the performance of the test.

### Statistical methods

A statistical analysis was performed using SPSS version 17.0 (SPSS Inc., Chicago, IL, USA). Pearson’s chi-square test for matched pairs was used to compare the performance of the two types of samples regarding the detection of HPV genotypes. Statistical significance was defined as *p* < 0.05.

## Results

The mean age of the 164 participants was 45.8 years. Among the women with normal cytology, the mean age was 50.5 years, while among those with abnormal cytology (LSIL or HSIL), the mean age was 41.1 years. Results from the cervical swab samples showed that 39.6% (65/164) were HPV DNA-positive (Table 1). In total, 11% (18/164) contained multiple HPV genotypes. The most common HPV genotypes detected were HPV16 (12 samples) and HPV18 (8 samples). Thus, 12.2% (20/164) contained either HPV16 or HPV18. In the normal cytology group, 11 of the 95 samples (11.6%) were HPV DNA-positive. In contrast, the LSIL and HSIL groups had 35 (10.0%) and 19 (100.0%) HPV DNA-positive samples, respectively.

**Table 1:** Detection of HPV genotypes and concordance between cervical swab and urine samples.

Cytology	Specimen (% positive)				Concordance (percentage)
	Any HPV positive		HPV16&18		
	Cervical swab	Urine	Cervical swab	Urine	
Normal (*N* = 95)	11 (11.6)	10 (10.5)	1 (1.1)	4 (4.2)	68 (71.6)
LSIL (*N* = 50)	35 (10.0)	35 (10.0)	11 (22.0)	13 (26.0)	31 (62.0)
HSIL (*N* = 19)	19 (100.0)	8 (42.1)	8 (42.1)	3 (15.8)	8 (70.5)
Total (*N* = 164)	65 (39.6)	53 (32.3)	20 (12.2)	20 (12.2)	107 (65.2)

For the urine samples, 32.3% (53/164) were HPV DNA-positive (Table 1), of which 7.9% (13/164) had multiple HPV genotypes. The most commonly detected HPV genotypes were HPV18 (17 samples) and HPV16 (four samples). In total, 12.2% (20/164) contained HPV16 or HPV18. In the normal cytology group, 10 of the 95 samples (10.5%) were HPV DNA-positive. In contrast, the LSIL and HSIL groups had 35 (10.0%) and 8 (42.1%) HPV DNA-positive samples, respectively.

**Table 2:** Studies of human papillomavirus DNA detected in paired urine and cervical samples from females of all ages.

Author	Country	HPV detection assay	Age, years range	Total sample size	Lesion/HPV types	Sensitivity (%)	Specificity (%)	Concordance (%)
Strauss et al. (1999)	UK	PCR with MY and GP primers	16–57	144	All/any type	76.4	73.3	75.7
Daponte et al. (2006)	Greece	In house type-specific primers and commercial	N/A	77	All/HPV16/18	70.3	100.0	85.7
Gupta et al. (2006)	India	In house L1 consensus primers	N/A	30	All/any type	100.0	100.0	100.0
Cuschieri et al. (2011)	UK	HPV INNO–LiPA	16–25	90	All/any type	90.5	67.6	59.8
Nilyanimit et al. (2013)	Thailand	Electrochemical DNA chip	27–61	116	All/HR-HPV	64.3	100.0	75
Bernal et al. (2014)	Spain	Cobas 4800HPV test	21–65	125	All/any type	90.5	85	88
Hagihara et al. (2016)	Japan	Anyplex II HPV28	19–58	240	All/any type	68.4	99.9	98.4
This study	Thailand	Hybribio GenoArray	19–69	164	All/any type	56.5	70.6	65.2

**Notes.**

N/A not applicable

We next compared HPV detection efficacy between the standard Pap smear samples and the urine samples. Comparison between the cervical swab and urine specimen resulted in the overall concordance of 65.2% (107/164) (Table 1). In the normal cytology group, the concordance was 71.6% (68/95). In the abnormal cytology group, the concordance was 56.5% (39/69). Using the Pap smear results as reference, the sensitivity and specificity of the urine-based HPV GenoArray Detection Kit were 56.5% and 70.6%, respectively. However, these results were lower than other studies (Table 2). The positive and negative predictive values were 53.8% (95% CI [41.9–65.4]) and 72.7% (95% CI [63.2–80.5]), respectively.

For multiple HPV infections, the cervical swab-based assays were able to detect more HPV genotypes in each sample. However, in the normal cytology group, for each pair of biospecimens, the most common number of genotypes per sample was one. Similarly, in the abnormal cytology group, 36 of the 69 cervical swab samples (52.2%) and 31 of the 69 urine samples (44.9%) had a single genotype (Table 3).

**Table 3:** Number of HPV genotypes detected using the HPV GenoArray assay.

No. of HPV genotypes detected	Normal (*N* = 95)		Abnormal (*N* = 69)	
	Cervical swab	Urine	Cervical swab	Urine
0^a^	84 (88.4)	85 (89.5)	15 (21.7)	26 (37.7)
1	11 (11.5)	9 (9.4)	36 (52.2)	31 (44.9)
2	–	1 (1.1)	14 (20.3)	7 (10.1)
≥3	–	–	4 (5.8)	5 (7.3)

**Notes.**

a Samples were HPV DNA-negative.

An analysis of the urine samples and a second analysis of the cervical swab samples showed that the differences in the overall HPV detection rate between the normal and abnormal cytology groups were not significant (*p* > 0.05).

## Discussion

Despite the benefits offered by the Pap test, screening attendance remains low (Gakidou, Nordhagen & Obermeyer, 2008), while the estimated incidence of invasive cervical cancer remains high (Leyden et al., 2005). In Thailand, 25–38% of women aged 30–65 years have had only one Pap test (Sriamporn, Khuhaprema & Parkin, 2006). When cervical testing for HPV is required, these results suggest that urine sample collection provides an alternative non-invasive sampling method for monitoring HPV infection in women. In a previous study, the overall percentage agreement between HPV detection in urine and cervical samples was 88% using the Cobas 4800 HPV test (Bernal et al., 2014), 75% using an electrochemical DNA chip (Nilyanimit et al., 2013) and, in this study, the percentage was 65.2%. The undeniable advantage in testing urine sample is its acceptance and convenience for the patients, although better results are obtained with first urine in the morning (Vorsters et al., 2014). However, the results must be interpreted with caution owing to variations among the studies in terms of the participant characteristics, surrogate nature of using cervical HPV detection to screen for cervical disease, and lack of standardized urine testing methods.

Urine sample assays cannot be used to detect all of the genital HPV infections, but these assays provide an alternative for use in epidemiological surveys in which invasive sampling is difficult to perform. Under these circumstances, testing urine for HPV DNA offers a distinct advantage (Prusty et al., 2005). Previous studies have compared HPV detection rates between cervical and urine samples in order to evaluate the ability of urine-based assays to detect the prevalence of HPV independently of cervical cytology assays (Daponte et al., 2006; Munoz et al., 2013). Evidence suggests that the sensitivity of urine testing for HPV 16 and 18 was higher for participants with cervical cancer (88.8%) than for those with high- and low-grade lesions (Daponte et al., 2006). However, since our samples comprised more of normal than abnormal cytology, proportion of HPV16-positive samples are thus admittedly lower in this study. The larger-scale HPV genotyping for cervical cancer screening in China showed the most common high risk HPV genotypes in women population worldwide were HPV16, 18, 31, 58, 52, 51 and 33, however frequencies varied by region (De Sanjosé et al., 2007). In contrast, some regions showed that HPV52 is higher detection than HPV16 because of the geographical and biological interaction between HPV genotypes and host immunogenic factors (De Sanjosé et al., 2007; Ye et al., 2010). Alternatively, it is possible that certain urine samples yield low-efficiency amplification due to the presence of inhibitory substances in the urine or HPV DNA loss during processing (Brinkman et al., 2004).

The HPV DNA analysis of urine samples needs to be developed further before a urine-based assay can replace the Pap smear test. It is possible that a greater amount of urethral cells in the urine samples helped to increase the sensitivity of the test. An analysis of the urine samples and a second analysis of the cervical swab samples showed that the differences in the overall HPV detection rate between women with normal and abnormal cytology were not significant (*p* > 0.05). This result suggests that urine represents a viable substitute for cervical swabs. However, the urine samples should be optimized by preventing DNA degradation during extraction and storage, recovering cell-free HPV DNA in addition to cell-associated DNA, processing a sufficient volume of urine, and collecting the first portion of the urine stream in the morning (Vorsters et al., 2014).

Using traditional cytological analysis, it is difficult to determine accurate screening results for HPV-associated anogenital tumors. Therefore, HPV genotyping is an alternative screening method to be used in combination with traditional cytology for identifying patients at high risk of developing squamous cell carcinoma (Saslow et al., 2012; WHO, 2013). Nowadays, there are many HPV genotyping techniques for detecting HPV DNA, such as PCR, real-time PCR, restriction fragment length polymorphism (RFLP), Hybrid Capture, and Linear Array (Bernard et al., 1994; Cox et al., 1995; Castle et al., 2008). However, PCR and real-time PCR need specific expensive equipment (such as a thermal cycler), and these methods have not yet become common procedures in hospital laboratories (Hagiwara et al., 2007). This study used the HPV GenoArray Diagnostic Kit for HPV genotyping, which is a commercial kit that has recently been started to be used, especially in China (Liu et al., 2010). The results from the HPV GenoArray assay used in this study were a percentage-point (39.6%) higher compared to the results from a previous survey of Thai women (7.6%) (Chansaenroj et al., 2010) and one of Japanese women (22.5%) (Onuki et al., 2009). The higher percentage may be due to the small number of participants in our study sample.

The Linear Array HPV Genotyping Test has been widely used as a standard reference method for evaluating new methods. However, the HPV GenoArray Diagnostic Kit is an alternative technique for studies conducted in resource-limited laboratories because the cost of the HPV GenoArray Diagnostic Kit is lower than that of the Linear Array HPV Genotyping Test and the hybridization time is also lower (Li et al., 2012; Liu et al., 2010). Moreover, the HPV GenoArray assay can distinguish and identify HPV 52, which is one of the most common high-risk HPV genotypes in women in eastern and southeastern Asia (Sukvirach et al., 2003; Takehara et al., 2011).

In conclusion, the HPV GenoArray assay is an alternative for HPV genotyping using both cervical swab and urine samples, the latter of which is an alternative for women declining to undergo Pap smears. Although it is not the gold standard, utilization of this method is expected to will be increase the number of women who undergo cervical cancer screening.

##  Supplemental Information

10.7717/peerj.3910/supp-1 Data S1 Comparison data between urine and cervical swab samples of HPV genotyping

## Additional Information and Declarations

### Data Availability

The following information was supplied regarding data availability: The genotyping data comparison between urine and cervical swab samples has been provided as a Data S1.
